# Electrocardiographic and biochemical profile of acute coronary syndrome in hospitalized COVID-19 patients

**DOI:** 10.6026/973206300212065

**Published:** 2025-07-31

**Authors:** Sandeep Aharwar, Rajat Kumar Tuteja, Pushpendra Singh Sengar, Dhruv Chowda

**Affiliations:** 1 Department of Medicine, Atal Bihari Vajpayee Government Medical College, Vidisha, Madhya Pradesh, India; 2 Department of General Medicine, Dr. S.N. Medical College, Jodhpur, Rajasthan, India; 3 Department of Medicine, Bundelkhand Medical College & Hospital, Sagar, Madhya Pradesh, India; 4 Department of Medicine, LN Medical College & JK Hospital, Bhopal, Madhya Pradesh, India

**Keywords:** COVID-19, acute coronary syndrome (ACS), ST-elevation myocardial infarction (STEMI), non-ST elevation myocardial infarction (NSTEMI), cardiac biomarkers, inflammation, mortality, D-dimer, CRP, Troponin-T

## Abstract

COVID-19 has been associated with the occurrence of acute coronary syndromes (ACS) and the medical and scientific communities consider the virus to put acute coronary syndromes (ACS) at risk because of its inflammatory and thrombotic effects. In a cross-sectional study of 100 hospitalized patients with COVID-19, 26 percent had ECG-confirmed ACS (16 percent STEMI/10 percent NSTEMI). The highest rates of elevated inflammatory and cardiac biomarkers occurred among older people, as well as diabetic and hypertensive patients. STEMI was substantially correlated to age >60 and increased fatality. It has become vital to detect cardiac involvement of COVID-19 at an early stage to diminish negative outcomes.

## Background:

The development of Coronavirus Disease 2019 (COVID-19) due to the novel severe acute respiratory syndrome coronavirus 2 (SARS-CoV-2) conditions has given rise to a range of clinical complications, that is, beyond the respiratory system [1]. Although most of the patients have mild or moderate symptoms, quite a high proportion of patients show severe symptoms, which involve cardiovascular issues, including myocardial damage, arrhythmias and acute coronary syndromes (ACS) [2, 3]. Systemic inflammation, endothelial dysfunction, cytokine storm and procoagulant state act as the underpinnings of the COVID-19 association with cardiovascular complications and generate plaque instability and the formation of thrombus [4]. The effects can worsen pre-existing cardiovascular disorders or trigger acute coronary events in people who never had them in the first place [5]. In addition, hypoxia caused by COVID-19, an elevated metabolic load and the direct invasion of the cardiomyocyte by the virus exert additional pressure on the cardiovascular system [6]. In determining patients with myocardial injury, electrocardiography and cardiac biomarkers are crucial. Higher troponins, CPK-MB and indicators of inflammation, including D-dimer, CRP, ferritin and LDH, are highly recorded and can be associated with poor clinical outcomes [7, 8]. Other risk factors that may alter the development of ACS in the case of COVID-19 are age, sex and comorbidities, including diabetes and hypertension [9, 10].

Depiction of COVID-19 pneumonia and ACS, including dyspnea and chest pain, is very similar, which contributes to the underestimation and untimely treatment of cardiac incidents. Serial biomarker measurements as well as ECG monitoring are therefore crucial in early identification and risk stratification of such Patients [11, 12]. These are suppression of the myocardium by cytokines, a rise in the sympathetic tone, thrombosis of the microvasculature and direct invasion of the myocardium through angiotensin-converting enzyme 2 (ACE2) receptors, which are extremely abundant in the heart [13]. Such multifactorial processes result in a wide spectrum of clinical manifestations, including myocarditis and arrhythmias, as well as thromboembolic symptoms and infarction-like conditions, which require a multidisciplinary treatment of the affected patients [14]. The previous knowledge of particular ECG patterns and the biochemical trends with COVID-19 patients that result in the development of ACS could help contribute to prompt diagnosis and an individual approach to treatment. There is also the potential prognostic usefulness of determining correlations between inflammatory markers and cardiac injury. During the pandemic, when the healthcare systems are overloaded, early identification of at-risk patients must be crucial to enhance survival outcomes. Therefore, it is of interest to evaluate the clinical, biochemical and electrocardiographic attributes that distinguish patients with acute coronary syndromes among individuals with COVID-19 hospitalization.

## Materials and Methods:

This was a cross-sectional observational study conducted in the Department of Medicine at Sri Aurobindo Institute of Medical Sciences (SAIMS), Indore, over a period spanning from April 2021 to September 2022.

## Study population:

A total of 100 adult patients diagnosed with COVID-19 infection were enrolled in the study. Inclusion criteria included:

[1] Confirmed SARS-CoV-2 infection using RT-PCR or rapid antigen testing,

[2] Hospitalized status during the study period,

[3] Age ≥ 18 years and

[4] Willingness to participate with informed consent.

Patients were excluded if they had pre-existing coronary artery disease or presented with ECG changes before COVID-19 diagnosis.

## Data collection:

After obtaining ethical clearance, demographic information (age, sex), clinical symptoms and comorbidities (such as diabetes, hypertension and anemia) were documented. Each participant underwent a 12-lead electrocardiogram (ECG) and findings were classified as:

[1] ST-Elevation Myocardial Infarction (STEMI)

[2] Non-ST Elevation Myocardial Infarction (NSTEMI)

[3] Normal ECG

Laboratory investigations were conducted using standard protocols and included:

[1] Complete Blood Count (CBC): Hemoglobin, total leukocyte count and platelet count.

[2] Inflammatory Markers: C-reactive protein (CRP), D-dimer, lactate dehydrogenase (LDH) and serum ferritin.

[3] Cardiac Enzymes: Creatine phosphokinase-MB (CPK-MB) and Troponin-T (TROP-T).

## Outcome measures:

The primary outcome was the detection of ACS (STEMI or NSTEMI) based on clinical, biochemical and ECG evidence. Secondary outcomes included the association of biochemical markers with ACS subtypes and overall in-hospital mortality.

## Statistical analysis:

All collected data were compiled and analyzed using SPSS version 20. Descriptive statistics were used to summarize demographic and clinical features. Associations between categorical variables were tested using Chi-square and Fisher's exact test, with a significance level of p< 0.05 considered statistically significant.

## Results:

A total of 100 patients diagnosed with COVID-19 were included in the study. The mean age of participants was 61.50 ± 14.36 years, with 60% (n=60) aged above 60 years. Male predominance was observed, with 68% (n=68) being males and 32% (n=32) females. Most patients (63%) had hemoglobin levels below the normal range, while 53% presented with leukocytosis and 23% had thrombocytopenia. Electrocardiographic changes indicative of acute coronary syndrome (ACS) were noted in 26 patients. Of these, 16% were diagnosed with ST-elevation myocardial infarction (STEMI) and 10% had non-ST elevation myocardial infarction (NSTEMI). The remaining 74% had no significant ECG changes suggestive of ACS (Table 1 - see PDF). Among patients with STEMI, the majority were aged above 60 years (81.3%), which was statistically significant (p = 0.014). STEMI occurrence was also significantly linked to increased mortality, with 75% of STEMI patients not surviving hospitalization (p = 0.032) (Table 2 - see PDF). Regarding biochemical findings, elevated D-dimer levels were found in 77%, serum ferritin in 79%, CRP in 69% and LDH in 48% of patients. Cardiac markers were raised in a smaller proportion: CPK-MB in 16% and TROP-T in 26%. Mortality among the total sample was 20%, with a higher rate among those who had elevated cardiac markers and ECG-confirmed STEMI (Table 3 - see PDF).

## Discussion:

The paper brings out the relationship between Coronavirus disease 2019 (COVID-19) and acute coronary syndromes (ACS) by focusing on the clinical, biochemical and electrocardiographic characteristics of the admitted patients. The incidence of ACS observed in this group (26%) indicates that SARS-CoV-2 infection possibly provokes myocardial ischemia, either directly because of myocardial injury or by systemic inflammatory reactions. The mean age of the patients was 61.5 and most of the ACS cases fell in patients who were over 60 years old. This conforms to earlier studies on the fact that older patients are more susceptible to cardiovascular complications during COVID-19 infection as a result of endothelial dysfunction caused by age and comorbidities [1, 2]. Male patient prevalence in this study agrees with previous reports, showing that male sex is an independent risk factor for poor cardiovascular outcomes in COVID-19 patients [3, 4]. The present study identified STEMI (16%) as more common than NSTEMI (10%) incidence among the cases of ACS. The observation confirms the hypothesis that COVID-19 induces a hypercoagulable condition and predisposes to full coronary artery occlusion [5]. Thomas further confirms the presence of an inflammatory and prothrombotic picture of severe COVID-19 due to high D-dimer and CRP in most of the patients (77 and 69 percent, respectively) [6, 7]. The poor outcomes that have been attributed to these laboratory abnormalities in previously conducted studies make them significant prognostic factors [8, 9]. The level of cardiac biomarkers, including the elevation of Troponin-T and CPK-MB, was increased in a few patients, which was in line with the occurrence of myocardial injury. The existence of these elevations was more prevalent in patients who had STEMI or failed to survive, which reinforces the importance of biomarker monitoring in the COVID-19 treatment [10]. In a past finding, elevated levels of troponin have been linked to higher levels of mortality among hospitalized participants with COVID-19, regardless of the presence of coronary artery disease [11, 12]. Notably, the illness fatality rate was high in patients with STEMI in this trial, as 75 percent of them died as a result of clinical manifestations of associated COVID-based myocardial infarction. This mortality is comparable to the studies conducted by other tertiary care facilities in India and the world, where it is important to consider the priority of early cardiovascular risk evaluation in COVID-19 patients [13, 14]. Comorbid disease is common in ACS patients, with diabetes and hypertension being common. This supports past evidence that these comorbidities in COVID-19 patients worsen the cardiovascular stress associated with the disease and raise the likelihood of developing negative outcomes [15, 16-17]. Management of these chronic diseases is critical in reducing complications in case of SARS-CoV-2.

## Conclusion:

COVID-19 poses a significant threat to ACS and especially older comorbid patients. The evidence indicates the necessity of regular cardiac assessment and active monitoring of the inflammatory and thrombotic markers in hospitalized COVID-19 patients.
